# Special Issue: Bioactive Materials for Additive Manufacturing

**DOI:** 10.3390/ma16186129

**Published:** 2023-09-08

**Authors:** Radosław Wichniarek

**Affiliations:** Faculty of Mechanical Engineering, Poznan University of Technology, Piotrowo 3, 60-138 Poznan, Poland; radoslaw.wichniarek@put.poznan.pl

The Special Issue, entitled “Bioactive Materials for Additive Manufacturing”, aims to cover cutting-edge research regarding the production, characterization and application of bioactive materials that can be processed using additive manufacturing technology. Particular emphasis is placed on presenting research on new materials containing active ingredients that have not been previously processed additively or for which new additive manufacturing methods have successfully been applied. This Special Issue is dedicated to all types of bioactive materials, both of natural origin and synthesized.

Additive manufacturing, commonly known as 3D printing, comprises a collection of several dozen different manufacturing methods. The wide range of available materials for processing, as well as the ways in which individual additive manufacturing methods interact with the processed material, are two of the many reasons why additive manufacturing has found applications in almost every field of engineering. Many examples of practical applications of additive manufacturing can be found in the field of biomedical engineering. For many years, research has been conducted on surgical aids for surgeons and students, in addition to customized orthoses, prostheses, and implants [1,2]. Numerous examples from the scientific literature are not limited solely to human treatment but also extend to the veterinary field [3].

While previously, additively manufactured biomedical products could be post-processed by adding an additional bioactive material, in recent years bioactive materials have increasingly been used directly during the additive manufacturing stage, most commonly as ingredients in the processed material. These bioactive ingredients can be both natural and synthetic [4,5]. The most commonly encountered practical examples found within the literature regarding the application of bioactive materials in additive manufacturing primarily pertain to implants, tablet-form medications, and transdermal drug delivery systems [6,7,8].

Although the field of using additive manufacturing for bioactive materials is expanding quickly, there are still numerous technical problems and obstacles that need to be solved. Some bioactive materials may be sensitive to chemicals and high temperatures, which can affect their stability and degrade their properties [9]. Synthetic materials used in 3D bioprinting may have poor biocompatibility, toxic degradation products, and a lack of bioactivity [10]. To fully exploit the promise of additive manufacturing in the biomedical industry, multidisciplinary research is required. The combination of research in bioactive materials with additive manufacturing holds great promise, particularly for patient-specific therapeutic applications [11].

Topics of interest for this Special Issue include, but are not limited to, the following: the characterization and properties of bioactive materials, the study of methodologies for the preparation of bioactive materials, storage and processing using various methods of additive manufacturing, and the effects of bioactive materials on organisms. Furthermore, significant contributions can be made through studies on practical examples of the application of bioactive materials, whether as additively manufactured products or as enhancements of existing products that were previously exclusively manufactured using conventional processing techniques.
